# Ligaments of the os trigonum: an anatomical study

**DOI:** 10.1007/s00276-021-02694-w

**Published:** 2021-02-07

**Authors:** Paweł Szaro, Khaldun Ghali Gataa, Mateusz Polaczek

**Affiliations:** 1 Department of Radiology, Institute of Clinical Sciences, Sahlgrenska Academy, University of Gothenburg, Gothenburg, Sweden; 2 Department of Descriptive and Clinical Anatomy, Medical University of Warsaw, Warsaw, Poland; 3 Department of Musculoskeletal Radiology, Sahlgrenska University Hospital, Göteborgsvägen 31, 431 80 Gothenburg, Sweden; 4 Medical University of Warsaw, Warsaw, Poland

**Keywords:** Os trigonum, Connections, Ligament, Ankle, Paratenon

## Abstract

**Purpose:**

The aim of the study was to examine the ligaments of the os trigonum.

**Methods:**

The ankle joint magnetic resonance imaging (MRI) of 104 patients with the os trigonum (experimental group) and 104 patients without the os trigonum (control group) were re-reviewed. The connections of the os trigonum and posterior talofibular ligament (PTFL), the fibulotalocalcaneal ligament (FTCL), the paratenon of the Achilles tendon, the posterior talocalcaneal ligament (PTCL), the osteofibrous tunnel of the flexor hallucis longus (OF-FHL) and the flexor retinaculum (FR) were studied.

**Results:**

The os trigonum is connected to structures. The posterior part of the PTFL inserted on the os trigonum in 85.6% of patients, whereas in all patients in the control group, the posterior part of the PTFL inserted on the posterior talar process (*p* < 0.05). The connection of the PTCL was seen in 94.2% of patients in the experimental group, while it was seen in 90.4% of patients in the control group (*p* > 0.05). The connection to the FTCL in the experimental group was 89.4%, while in the control group, it was 91.3% (*p* > 0.05). The communication with the paratenon was seen more often in the control group compared to that in the experimental group (31.7% vs. 63.8%, *p* < 0.001). The FTCL was prolonged medially into the FR in 85.6% of patients in the experimental group and in 87.5% of patients in the control group (*p* > 0.05). The flexor hallucis longus (FHL) run at the level of articulation between the os trigonum 63.5% and the posterior process of the talus 25% and less often on the os trigonum 11.5%.

**Conclusion:**

The os trigonum is connected with all posterior ankle structures and more connections than previously reported.

## Introduction

The os trigonum is the second most common accessory bone present in the foot and is often the cause of posterior ankle impingement [34]. Different authors provide various frequencies (2–13%) of the occurrence of the os trigonum [1, 2, 11, 34]. However, in some clinical studies, the frequency is higher (20–30%) [10, 31, 34]. The anatomy of the ligaments of the os trigonum have not been investigated in detail, while the ligaments of Kager’s fat pad (KFP) [7] at the level of the posterior process of the talus have been described in detail previously [27]. PTFL inserts on the os trigonum and the posterior process of the talus [8]. At the level of the posterior process of the talus, the ligaments of KFP integrate. From the lateral side, the superior peroneal retinaculum (SPR) and FTCL unite. Occasionally, a projection from the SPR to the anterior talofibular ligament (ATFL) is visible. PTCL joins the calcaneus centrally and posteriorly to the posterior process of the talus [9]. The paratenon receives a projection from the FTCL. The connection to FR and OF-FHL is noticed on the medial side [26, 27]. There is no research regarding the ligaments of KFP when the os trigonum is present.

The os trigonum is related directly to the flexor hallucis longus (FHL) and KFP. The posterior part of the talofibular ligament inserts on the os trigonum [8]. Other ligaments of the posterior process of the talus occur variably [27]. PTCL is present in about 80% of cases and connects the posterior process or os trigonum with the superior and medial part of the calcaneus. The formation of the letter “V” can seldom be seen, because the ligament is formed from two fascicles originating from the medial and lateral tubercles on the posterior process of the talus, with the apex on the calcaneus [9, 17]. When the os trigonum is present, the PTCL is usually formed from one fascicle; some authors call it the trigonocalcaneal ligament [17].

FTCL is a variably occurring ligament [18], which together with the PTFL and calcaneofibular ligament inserts on the lateral malleolus [17, 27]. It is divided into two thin fibrous laminae. The superomedial lamina attaches onto the lateral tubercle of the posterior process of the talus, while the inferolateral lamina runs to the calcaneus [17, 27].

It cannot be unequivocally ruled out that the degree of mobility of the os trigonum influences the occurrence of impingement symptoms. During plantarflexion, the os trigonum and surrounding soft tissue may become impinged between the posterior distal surface of the tibia and superior surface of the calcaneus [33]. Direct impingement of soft tissue or bony elements may result in structural changes of the os trigonum, such as bone marrow oedema (BME) as an indicator of the symptomatic os trigonum [12, 28]. It is not clear why some accessory bones become symptomatic and others do not [10, 28].

The aim of our study was to investigate the detailed anatomy of ligaments of the os trigonum on MRI.

## Material and methods

### Inclusion criteria

Retrospective analysis of the magnetic resonance imaging (MRI) of patients with a recognized os trigonum.

The MRI examinations were done between January 1, 2011, and May 31, 2018. In the study, we included consecutive patients with the presence of an os trigonum, while in the control group, we included the same number of consecutive patients without an os trigonum.

We included only patients with complete clinical data and MRI examination protocols, including at least: T1 coronal (with or without fat saturation), T2 fat saturation coronal and axial, sagittal short-TI inversion recovery (STIR) and proton density axial (with or without fat saturation). All MRI examinations were performed using a 1.5 T scanner and were reviewed by two observers, and the final decision was made by consensus. MRI parameters were as follows: the sagittal and axial fast spin-echo (FSE) sequences had a field of view (FOV) of 14 × 14 cm and a slice thickness of 3 mm without spacing. The coronal sequences had a FOV of 10 × 8 cm. Matrix in axial plane 256 × 218, in coronal plane 256 × 230 in sagittal plane 320 × 272. The echo time in PD 20 while in T2-weighted FSE was 60 ms. The rage of the repetition time in PD was 2000–5000 ms, while in T2-weighted 3000–5000 ms.

### Exclusion criteria

We excluded all patients with a history of previous fracture (22 cases excluded), obvious abnormality within KFP (two cases excluded), abnormality in one of the examined structures (19 cases excluded) and remaining orthopedic hardware due to the possible artifacts (19 cases excluded).

After application of the inclusion and exclusion criteria, 104 MRIs of patients with an os trigonum were included in the experimental group and the same number of patients without an os trigonum were included in the control group.

The structures included in the study were the os trigonum, posterior talar process, PTFL, PTCL, FTCL, OF-FHL, paratenon and FR.

### MRI review

First, the os trigonum was found on the horizontal and sagittal scans. Second, on axial images the PTFL, OF-FHL, paratenon and FTCL were assessed. Then, on sagittal section, the connection of the os trigonum to the PTCL was examined. The presence of a connection was recognized when the direct communication of the os trigonum smoothly merged into an examined structure. Observations were confirmed on at least one or more planes.

### Statistical method

To prove if there was a significant difference in the occurrence of the connections in the group with an os trigonum and control group, *p* < 0.05 was considered statistically significant.

The Local Ethics Committee approved the study and the need for informed consent was waived (Number 2020-06177).

## Results

The experimental group included 57 females and 47 males (51 right and 53 left), with an average age of 35.8 ± 9.9 years (range 18–55 years). The control group included 53 females and 51 males (55 right and 49 left), with an average age of 37.5 ± 10.4 years (range 19–65 years). No significant differences in the age or gender distribution were noticed between the two groups (*p* > 0.05).

**Fig. 1:**
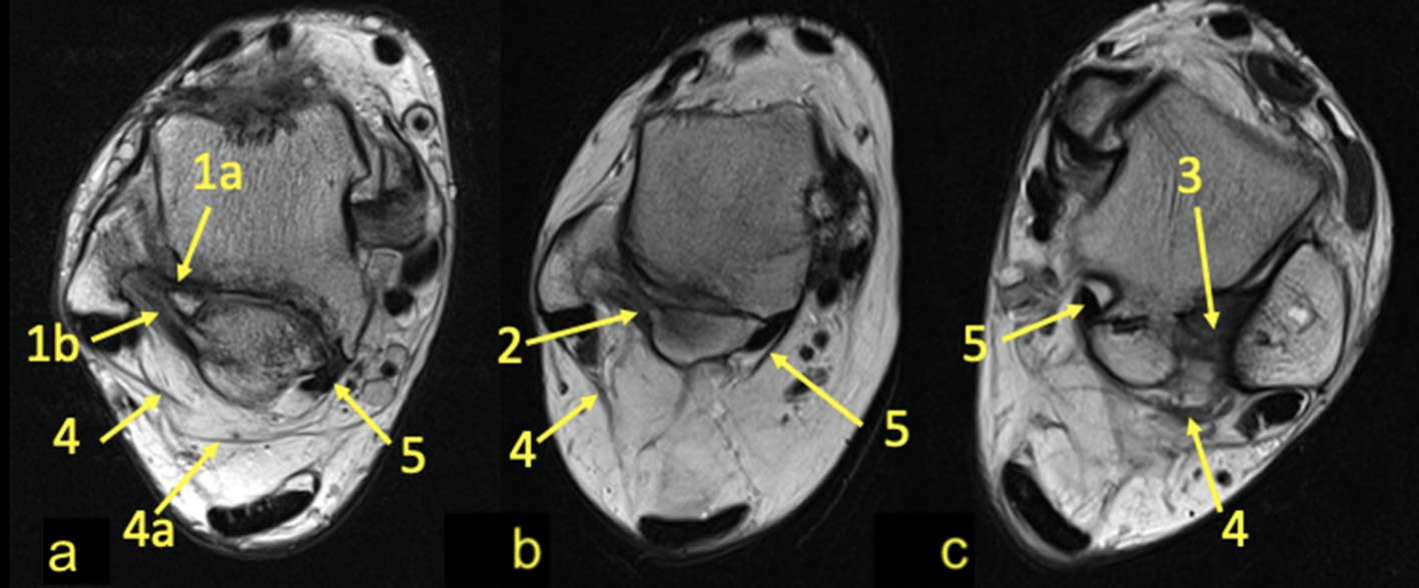
Three variants of the insertion of the PTFL. PD-weighted—**a**–**c** 1a—anterior part of the PTFL inserts on the talus; 1b—posterior part of the PTFL inserts on the os trigonum; 2—the PTFL inserts only on the os trigonum; 3—the PTFL inserts only on the talus; 4—the FTCL; 4a—the inferolateral lamina of the FTCL; 5—the FHL runs directly on the articulation of the os trigonum

We found connections between the os trigonum and PTFL, PTCL, OF-FHL, paratenon and FTCL, and FR both in the experimental and control group (Table 1). The connections have the form of narrow, partly communicating fiber bands in both groups. The ligament most commonly connected to the os trigonum was the PTFL (Table 1 and Fig. 1). In most cases of the experimental group, the posterior part of the PTFL inserted on the os trigonum (*n* = 89, 85.6%), while in 10 (9.6%) cases, the whole PTFL inserted on the os trigonum (Fig. 1). In five (4.8%) cases of the experimental group, the PTFL inserted only on the posterior process of the talus, and no attachment to the os trigonum was noticed (Fig. 1). In all cases in the control group, the PTFL inserted on the posterior process of the talus (Table 1).

**Table 1:** Connections of the os trigonum and control group

	FTCL	PTCL	OF-FHL	Paratenon lateral	Paratenon medial	FR	Node-like structure
Os trigonum	93	98	44	33	6	89	41
	89.4%	94.2%	42.3%	31.7%	5.8%	85.6%	37.5%
Control group	95	94	55	67	31	91	43
	91.3%	90.4%	52.4%	63.8%	29.5%	87.5%	41.3%
*p*	> 0.05	< 0.001	> 0.05	< 0.001	< 0.01	> 0.05	> 0.05

Connections to the PTFL, PTCL, FTCL, OF-FHL, paratenon and FR

*PTFL* posterior talofibular ligament, *PTCL* posterior talocalcaneal ligament, *FTCL* fibulotalocalcaneal ligament, *OF-FHL* osteofibrous tunnel of the flexor hallucis longus, *paratenon med.* medial part of the paratenon*, paratenon lat.* lateral part of the paratenon, *FR* flexor retinaculum

**Fig. 2:**
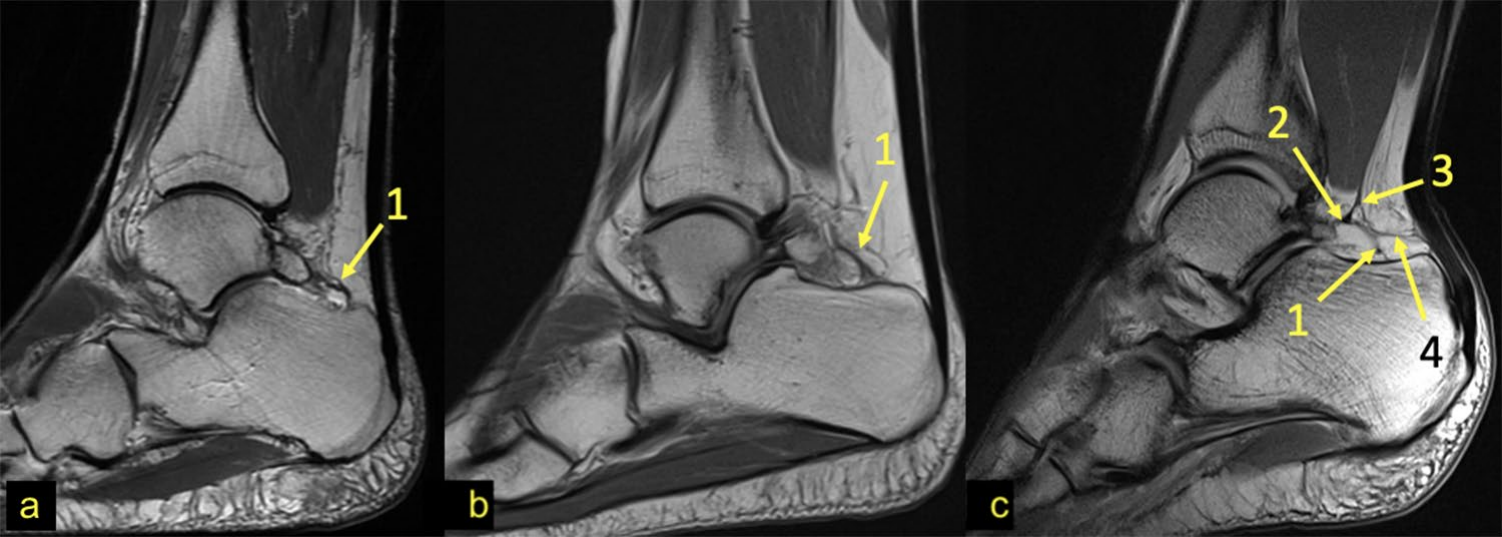
Connections to the PTCL and node-like structure. Three different patients, sagittal PD sequence. **a** and **b**—the PTCL (1). **c**—the PTCL (1), the presence of a *node-like structure* (2) from which connections radiate to the FHL (3) and KFP (4)

**Fig. 3:**
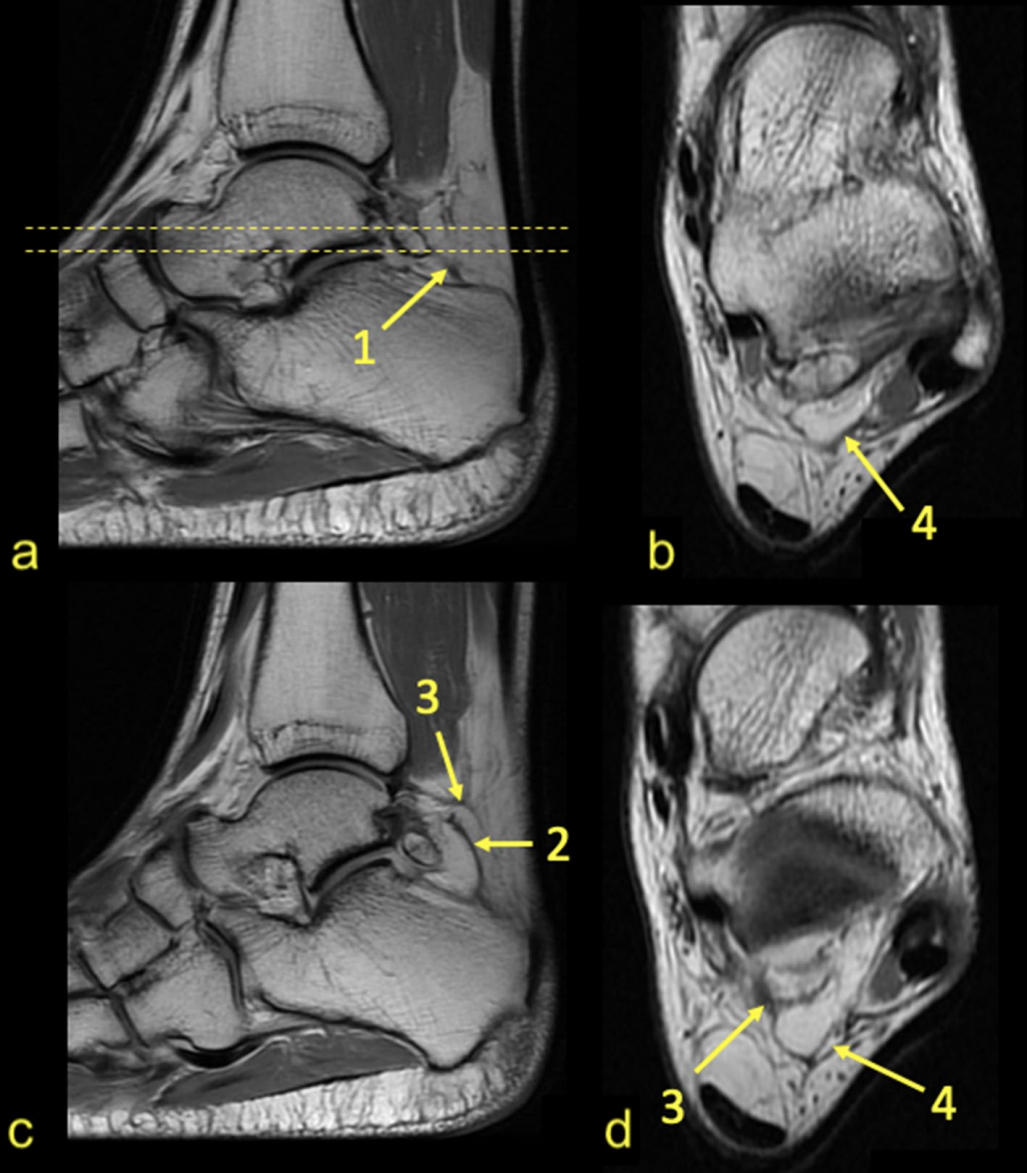
Connections to the PTCL. PD sequences for the same patient—**a**–**d**. The direct connection between the os trigonum and calcaneus via the PTCL, which branches in the fascicle to the calcaneus (1) and to the medial tubercle on the posterior process of the talus (2). The connections via the *node-like structure* (3) to the calcaneus (2) and to the superior peroneal retinaculum by means of the FTCL (4)

The PTCL was identified in 98 (94.2%) cases of the experimental group, of which in 54 (51.9%) cases inserted on the os trigonum, while in 44 (42.3%) cases, it inserted on the posterior process of the talus (Fig. 2). No clear PTCL was noticed only in 6 (5.8%) cases of the experimental group. In the control group, the PTCL was noticed in 94 (90.4%) cases (*p* < 0.001), while no clear ligament was seen in the remaining 10 (9.6%) cases. An accessory projection from the PTCL to the posterior outline of the joint capsule was noticed in 37 (35.6%) cases of the experimental group. Posterior to the os trigonum, a "fibrotic node-like structure" (Fig. 3) was visible in 41 (39.4%) cases vs. 43 (41.3%) cases in the control group (*p* > 0.05). The surrounding structures received fibrotic projections from fibrotic node-like structure (Figs. 2 and 3).

**Fig. 4:**
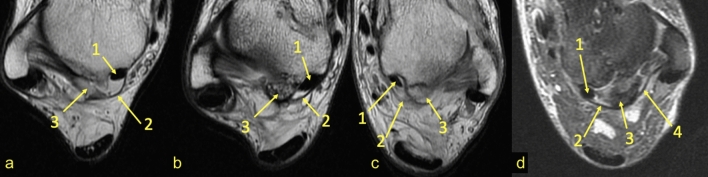
Connection to the flexor hallucis longus. Four different patients (**a**–**c** PD sequence, **d** PD with fat suppression). The flexor hallucis longus is in the osteofibrous tunnel, whose walls connect (2) with the os trigonum. The posterior part of the PTFL inserts on the os trigonum

**Fig. 5:**
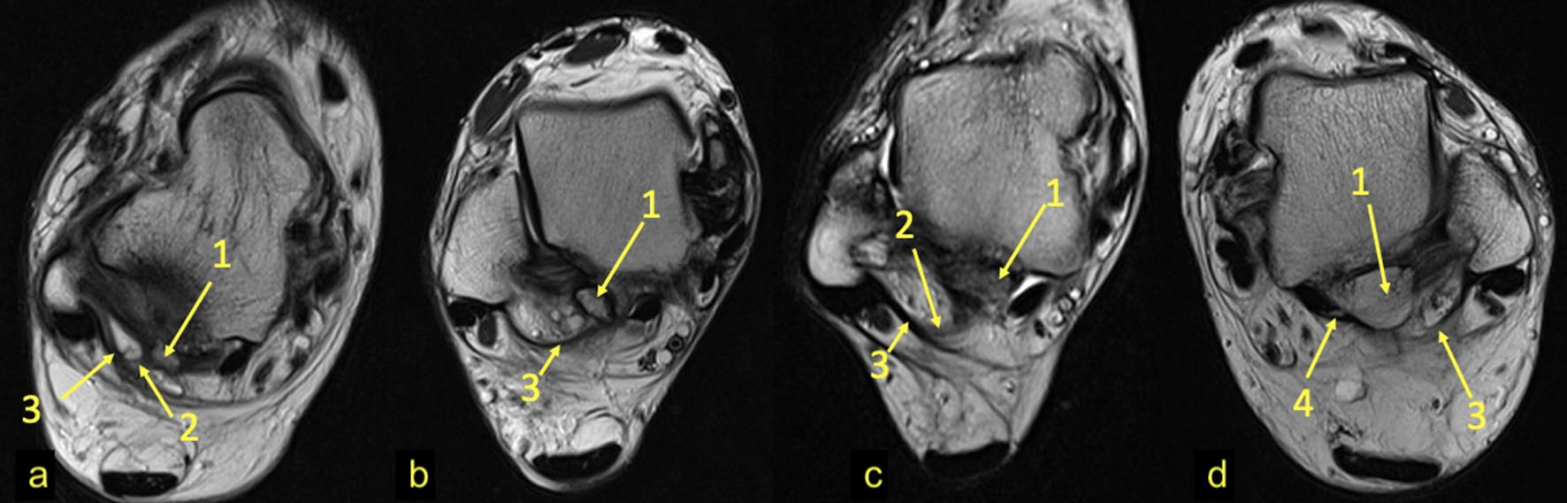
Connections to the FTCL. PD sequences for four different patients (**a**–**d**). The os trigonum (1) connects with the superior peroneal retinaculum by a band (2) from the fibulotalocalcaneal ligament (3). The connection between the os trigonum and osteofibrous tunnel of the flexor hallucis longus can be seen (4)

The connection to the OF-FHL (Fig. 4) was seen directly in 44 (42.3%) cases or through the fibrotic node (Fig. 3) mentioned above in 23 (22.1%) cases of the experimental group. FHL runs in most cases at the level of articulation between the os trigonum (*n* = 66, 63.5%), followed by the posterior process of the talus (*n* = 26, 25%) (Fig. 1) and less often on the os trigonum (*n* = 12, 11.5%) (Fig. 5).

The FTCL is a delicate thin fibrous lamina set in the frontal plane located between the os trigonum (n = 93, 89.4%) or the posterior process of the talus in the experimental group (*n* = 95, 91.3%), *p* > 0.05), medial and lateral malleolus and paratenon posteriorly. At the insertion on the lateral malleolus, the FTCL connected to the calcaneofibular ligament in 4 (3.8%) cases in the experimental group and in 9 (8.7%) cases in the control group (*p* < 0.05). The connection between the os trigonum and superior peroneal retinaculum via the FTCL was seen in 31 (29.8%) cases of the experimental group (Fig. 5). The FTCL prolonged medially into the FR in 89 (85.6%) cases in the experimental group and 91 (87.5%) cases in the control group (*p* > 0.05). The os trigonum was connected to the paratenon via the FTCL more often from the lateral side (*n* = 33, 31.7%) compared to that in the medial side (*n* = 6, 5.8%). The corresponding values in the control group were generally higher (*n* = 67, 63.8%, p < 0.001) for the lateral side compared to those in the medial side of the paratenon (*n* = 31, 29.5%, *p* < 0.01). The medial expansion of the FTCL connected with the plantaris tendon in *n* = 57 (54.8%) in experimental group in while in control group (*n* = 52, 50%, *p* > 0.05). The plantaris tendon was connected directly to the paratenon in (*n* = 19, 18.2%) cases in the experimental group and in (*n* = 23, 22.1%) cases in the control group (*p* > 0.05). The absence of a connection of the plantaris tendon with ligament projections included in the study was noticed in (*n* = 28, 26.9%) in the experimental group and (*n* = 29, 27.9%, *p* > 0.05) in the control group.

**Fig. 6:**
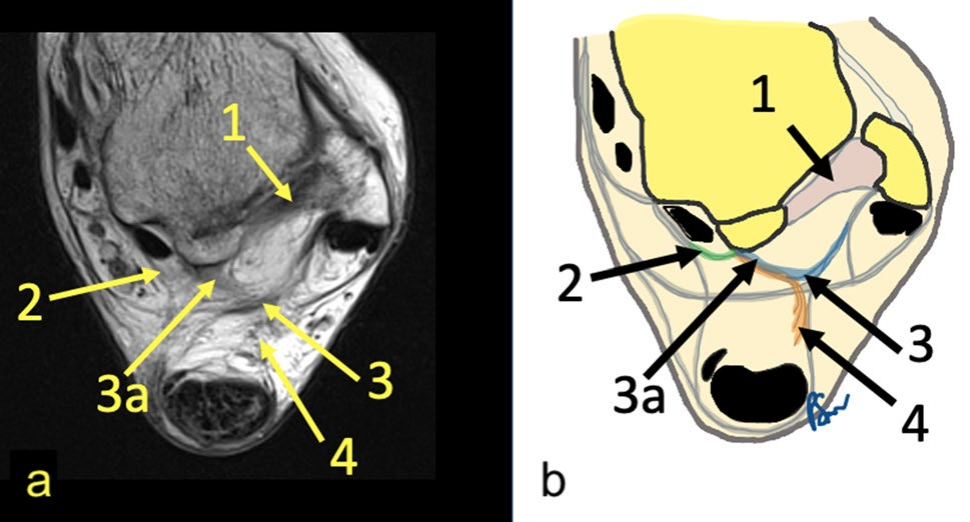
**a** Prominent connections of the os trigonum, PD sequence. **b** General map of the communications between different fibrotic structures at the level of the os trigonum. 1—PTFL, 2—connection to the osteo-fibrosus tunnel of the FHL, 3—FTCL, 3a—branch of FTCL to the os trigonum, 4—connection to the Kager’s fat pad

The connections of the os trigonum with neighboring structures are located within KFP (Fig. 6).

The most significant difference in the occurrence of connections between groups is visible in connections with the paratenon. The presence of the os trigonum is associated with less frequent connections to the paratenon. The plantaris tendon connects with FTCL in most cases of both groups. A less significant difference between the groups is visible in connections with PTCL, which is seen slightly more common in the experimental group. In the group of patients with the os trigonum, the connection to OF-FHL was somewhat less frequent than in the control group. In both groups, we noted node-like structures that are a connection centrum between different ligaments of the os trigonum. There was very little difference in the frequency of connections to FR between the groups. The most common variant of the os trigonum was a single bone (*n* = 102, 98.1%), followed by a bipartite os trigonum (*n* = 2, 1.9%).

## Discussion

To the best of our knowledge, there are neither radiological nor anatomical studies regarding connections of the os trigonum, and the current research is the first study. Our study revealed that the os trigonum is connected to the PTFL, FTCL, paratenon, PTCL, OF-FHL, and FR. The current study showed the differences in the frequency of connections of the os trigonum with other structures. The most significant differences were visible in connection to paratenon. The presence of the os trigonum associates with a lower incidence of connections to the paratenon. This may be because the plantar tendon was often associated with the medial projections of the FTCL.

According to previous reports, the posterior part of the PTFL inserts on the os trigonum [8]; however, we found a small number of cases where the os trigonum was present, but the PTFL only inserted on the talus. The presence of this rare variation was probably related to more cases in our group and a smaller diameter of the os trigonum revealed by us.

It was reported previously that the presence of the os trigonum was associated with a higher occurrence of the abnormality in the anterior talofibular ligament (ATFL) and PTFL [8], which may depend on the presence of the interconnections between the lateral ligaments inserting on the lateral malleolus [4, 25]. Mechanical strength and the presence of connections between the lateral ankle ligaments and other fibrous structures may lead to mutual dependence, resulting in concomitant abnormalities [3, 26, 27]. The os trigonum is probably more movable when both parts of the PTFL are inserted, leading to degenerative changes at the articulation, which in turn may cause microinjury of the FHL [8, 14]. In our material, the FHL runs mostly at the level of the articulation between the os trigonum and talus, which may predispose to injury.

KFP at the level of os trigonum is a kind of “coordination centrum” between different structures, which is similar to cases without an os trigonum that were reported previously [27]. At the level of the central part of KFP, in direct relation to the central part of the FTCL, the presence of a fibrotic “node-like” structure was noticed in both groups [27]. Due to its projections, the paratenon, FTCL, OF-FHL and FR are connected with the os trigonum or posterior process of the talus [7]. Mentioned above connections were slightly more common in the control group. The incidence of the “node-like” structure revealed in our study appears to be unrelated to presence of the os trigonum. The presence of ligaments and its projections in KFP can influence the presence of different compartments or functional subunits [7].

The PTCL originates on the lateral tubercle of the talus, which is called the os trigonum when it is not fused [8]. PTCL is a variable ligament occurring in most cases with and without os trigonum. The small difference statistically significant difference was found in the occurrence of the PTCL between our experimental and control groups; however, the discrepancy of the incidence found in the literature was probably due to great anatomical variability [9]. The PTCL is orientated in the sagittal plane, making the sagittal sections stretching between the posterior process of the talus or os trigonum and calcaneus assessable on the MRI [9, 27]. The function of the PTCL is not fully understood; however, due to its direction, the PTCL may be involved in the development of os trigonum syndrome, subtalar instability and flat foot [9, 19]. We found communication between the PTCL and articular capsule, which was not reported previously. The presence of this communication may influence the tension of the recess of the posterior ankle [17].

The connections to the paratenon are visible mostly on its lateral outline [7], which is probably due to the communication with the FTCL [18]. The connection with the paratenon was seen most often in our control group. The role of the plantaris tendon in the pathogenesis of tendinopathy has already been discussed in the literature [16]. However, the plantaris tendon's function is probably more complicated than before thought because of its connections. High variation of the plantaris tendon has been described before. There are variants where the plantaris tendon insertion divides, giving an anatomical basis for connections with the surrounding structures [13]. In most cases, the plantaris tendon has connections with the surrounding ligaments or their projections. Present of connections of the plantaris tendon to ligaments give the possibility of the influence of the ligaments' tension. The os trigonum was connected to the paratenon via the FTCL more often in control, indicating that the os trigonum changes the anatomical relations in KFP.

The development of the connections between ankle structures has not been examined before; therefore, it is difficult to unequivocally explain this observation. The connections revealed in our study have a fibrotic nature and are parts of the crural fascia system where the FR connects with the SPR via the FTCL, which also projects anteriorly and posteriorly to the os trigonum and paratenon, respectively [18]. These connections illustrate the integrative function of fascia thanks to the central localization of the FTCL and “node-like” structure [4, 27]. In this way, the crural fascia integrates the posterior ankle region without difference whether or not if the os trigonum is present.

The connection of the crural fascia with the paratenon or the OF-FHL illustrates the association between the muscle and fascia. It takes an anatomical background to understand traction-induced disorders described previously [24]. The most common connection of the paratenon is seen on its lateral side via the FTCL [18]. The presence of the os trigonum probably has an association with the frequency of connections. Pathology of the paratenon is a significant indicator of Achilles tendinopathy [21]. The paratenon is composed of a fibrous layer continuous with the crural fascia [22]; however, no previous research regarding connections between the paratenon and os trigonum is available. The paratenon has got an important role in the regeneration of the Achilles tendon thanks to its vessels possibly reaching the tendon through the fascial connections.

The FR integrates the deltoid ligament and spring ligament with other structures in the medial part of the ankle joint. We found no differences between the experimental and control groups regarding the presence of communication between the FTCL and FR. Both the FR and FTCL belong to the fascial elements; hence, they perform integrative functions thanks to the presence of projections to the surrounding structures and function in proprioception [23].

The connections revealed by us are projections of the crural fascia [18] present in the part of KFP that is related to the FHL [27]. The functionally different part of KFP adjacent to the Achilles tendon does not contain connections, except for the projections to the paratenon [5, 30]. Differentiated mobility and hence different distributions of the fibrotic bands in KFP [7] may change or modulate the traction of the os trigonum [6].

The presence of the os trigonum is related to non-fusion of the accessory ossification center with the posterior process of the talus, which may indicate that the presence of the accessory band can be developmentally conditioned [1]. Most of the cases described in the literature and revealed by us represent the os trigonum as a single bone separated from the posterior talar process by synchondrosis [8, 20].

To our knowledge, there are no anatomical studies describing the complete map of the fibrous connections in KFP when the os trigonum is present [27]. We are aware that carrying out anatomic research can be difficult due to the very fine structure of these bands. MRI shows high sensitivity in tissue differentiation, which allows us to distinguish even the tiniest fibrous bands against the background of the adipose tissue in KFP [29], thus, allowing anatomical studies of fascial elements. We believe that the use of 3D sequences could help to visualize ligaments of the os trigonum on MRI. The use of isovolumetric voxels enables image reconstruction in any plane. Because of the variable orientation of some ligaments, curved-planar reconstruction would help for better assessment. We see the need for anatomical studies regarding the os trigonum and KFP because of the development of new less invasive techniques of treatment for os trigonum syndrome [15, 32].

There are limitations to this study. Due to the retrospective character of the study, we could not influence the MRI protocol, as the examinations were performed because of clinical indications in a pre-defined protocol, with no surgical or anatomical correlation.

## Conclusion

The os trigonum connects with the posterior ankle structures by fibrotic connections that are part of the crural fascia and are located in KFP. The current study revealed connections of the os trigonum to the PTFL, FTCL, paratenon, PTCL, OF-FHL and FR. Connections to the paratenon were seen more often in our control group compared to those in our experimental group. Both in the presence and absence of the os trigonum a fascial integration system within KFP was noticed. The presence of connections revealed in our study may play an important role in the pathology of the paratenon.

## Data Availability

Yes.

## Code availability (software application or custom code)

Yes.
